# Discrete Element Damage Constitutive Model of Loess and Corresponding Parameter Sensitivity Analysis Based on the Bond Rate

**DOI:** 10.3390/ma18081726

**Published:** 2025-04-10

**Authors:** Hui Qi, Xiaoyan Liu, Haining Wang, Chao Hu

**Affiliations:** 1 School of Qilu Transportation, Shandong University, Jinan 250002, China; qihui0214@gmail.com; 2 Shandong Hi-Speed Group Co., Ltd., Jinan 250101, China; whm1992526@tom.com (H.W.); sdgsgcxxb@163.com (C.H.); 3 School of Mechanics and Civil Engineering, China University of Mining and Technology, Xuzhou 221116, China

**Keywords:** discrete element model, compacted loess, mechanical behavior, damage behavior, constitutive model

## Abstract

This study introduces a novel discrete element method (DEM) model for compacted loess, incorporating a bond rate parameter within a linear contact bond model to simulate constitutive damage behavior. This enhancement significantly improves the characterization of structural damage from repeated wet–dry cycles, offering a quantitative method for predicting damage progression. Unlike existing DEM models, our model directly uses a bond rate parameter to quantitatively describe inter-particle bond deterioration, reflecting reduced bonding strength due to pore structure development and the weakening effect of water. Rigorous calibration and validation were performed using comparative experiments. A key innovation is the systematic analysis of microscopic parameters (contact stiffness, friction coefficient, contact strength, and bond rate) and their impact on macroscopic mechanical behavior. Our findings show that decreasing the bond rate significantly reduces the macroscopic mechanical properties, providing valuable insights into the micro–macro relationship. We comprehensively evaluated prediction sensitivity to these parameters. This methodology offers a new perspective on using DEM for predicting crucial civil engineering material properties, providing a valuable reference for incorporating bond rate parameters into future modeling, particularly for long-term geotechnical material behavior under environmental degradation. The model’s accurate representation of wet–dry cycle effects on loess strength improves earth structure design and safety.

## 1. Introduction

In the field of geotechnical engineering, accurately capturing the progressive degradation of material strength is crucial, as it directly influences the reliability and safety of earth structures exposed to varying environmental conditions. The traditional investigation of soil behavior has been transformed by the discrete element method (DEM), which has significantly enhanced the simulation of particulate materials under diverse loading scenarios. At the core of DEM’s proficiency is the element model [1], which defines the interactions and bonding mechanisms between individual particles. However, a significant challenge remains: the lack of straightforward and robust approaches for modeling the loss of material strength in soils experiencing complex environmental stresses, such as wet–dry cycles [2,3,4,5]. This limitation hinders accurate failure prediction and lifespan management of geotechnical constructions.

The discrete element method (DEM) is a powerful numerical technique that has garnered widespread attention among researchers for its extensive applications in simulating and analyzing the mechanical response, damage, and failure behavior of various materials [6,7,8,9,10] and the failure of geo-structures [11,12,13]. Rougier et al. [14] introduced a combined plastic and discrete fracture deformation framework for finite-discrete element methods (FDEM) to simulate fracture in brittle materials like cementitious and rock-like materials. Meza-Lopez et al. [15] focused on modeling asphalt concrete fracture tests using DEM. Zhang et al. [16] compared the undrained behavior of granular media using the fluid-coupled discrete element method and the constant volume method. Chen et al. [17] investigated the collapse behavior of hard roofs in coal seams using DEM. Shi et al. [18] determined the coefficient of rolling friction of irregularly shaped maize particles using DEM. Nie et al. [19] examined the effect of local non-convexity on the critical shear strength of granular materials using DEM. Liu et al. [20] proposed a new discrete element-embedded finite element method for transient deformation, movement, and heat transfer in packed beds. Kuang et al. [21] developed a DEM-based approach for simulating particle breakage. De et al. [22] introduced a particle location-based multi-level coarse-graining technique for DEM simulations. Liu et al. [23] evaluated the composition criteria and characteristics of asphalt mixture skeleton main force chains using DEM. Li et al. [24] focused on modeling flexible membrane boundaries in drained/undrained triaxial tests using DEM. Tang et al. [25] conducted numerical simulations of the expansion process of soundless cracking demolition agents by coupling finite difference and DEM.

Coupling the discrete element method (DEM) with other numerical methods has become a research hotspot, overcoming limitations in simulating complex problems such as fluid–solid coupling and multi-physics coupling. Qu et al. [26] effectively simulated the failure process of a CFRD (concrete-faced rockfill dam) under seismic action using a two-dimensional DEM-FEM coupled method, quantifying the effects of seismic mitigation measures. Hu et al. [27] employed a multiscale FEM-DEM approach to study seepage-induced suffusion and slope instability, achieving good simulation results. Zhang et al. [28] used a three-dimensional CFD-DEM model to investigate scour around monopiles, revealing the crucial role of seepage-induced vertical drag force in the scour process. Li et al. [29] proposed a three-dimensional concurrent MPM-DEM scheme to simulate soil–rock mixed slopes, improving computational efficiency. Ahmadian et al. [30] used an LBM-DEM coupled method to simulate the fluid–solid coupling behavior of irregular particles. Chen et al. [31] effectively simulated the interaction between waves and permeable breakwaters using a CFD-DEM coupling method based on a semi-analytical model, improving the simulation accuracy. DEM coupling methods have significantly expanded the application range of DEM, providing effective approaches to solve complex engineering problems involving fluid–solid coupling and multi-physics coupling. Future research should focus on the applicability and limitations of different coupling methods and develop more efficient and accurate coupling algorithms.

In the prediction of damage and strength degradation, DEM has also been widely used in various studies. Yu et al. [32] utilized DEM to predict the strength and damage propagation of bolted composite lap joints under tension, showcasing the method’s potential in providing detailed local damage information at a micro-scale level. Similarly, Wang et al. [33] established a discrete model of cabbage seeds using DEM to simulate compression characteristics and crushing, demonstrating high consistency with physical experiments. Furthermore, Zhao et al. [34] and Novak et al. [35] employed DEM to analyze damage and failure in hydrate-bearing sediments and composite skin laminates, respectively. Zhao et al. [34] highlighted the advantages of DEM in accurately responding to the microstructure of samples, while Novak et al. [35] compared the results of discrete damage modeling tools with baseline models to predict damage initiation and propagation. In addition, Kosteski et al. [36] employed the lattice DEM to explore the fractal nature of scale effects in materials undergoing damage. The study emphasized the random nature of damage accumulation and its influence on the nonlinear behavior leading up to collapse. Dong et al. [37] and Liu et al. [38] focused on calibrating DEM parameters for rice bud seeds and corn straw cutting, respectively, using the Hertz–Mindlin model to simulate damage accumulation and crushing processes. Moreover, Peng et al. [39] applied a 3D DEM to predict the mechanical response of crumb rubber-modified asphalt pavements under traffic loads, considering temperature gradients and fatigue damage. Lastly, Zhu et al. [40] studied the evolution law of overlying strata structure in a stop using a “space–air–ground” integrated monitoring network and discrete element simulations, showcasing the versatility of DEM in analyzing structural changes over time. However, despite these advancements, current DEM methodologies still have limitations in terms of scalability, accurate representation of environmental factors, and long-term degradation predictions [41,42,43].

Existing DEM models often struggle to capture the nuanced effects of environmental degradation, such as the weakening of soil structure due to repeated wetting and drying cycles. These models frequently lack a direct, quantitative representation of the deterioration process. This study directly addresses these shortcomings by introducing a novel DEM model for compacted loess.

Our proposed model incorporates a “bond rate” parameter into a linear contact bond model. This parameter directly quantifies the reduction in inter-particle bonding strength due to factors such as pore structure development and the weakening effect of water. This innovative approach offers a significant improvement over existing methods, providing a more accurate and physically meaningful representation of damage progression under cyclic wet–dry conditions.

This innovation has the potential to significantly impact geotechnical engineering. By providing a more accurate and quantitative method for predicting damage progression, our model can improve the design and safety of earth structures, reducing the risk of failure. Furthermore, the systematic analysis of microscopic parameters and their influence on macroscopic behavior provides valuable insights into the micro–macro relationship in loess, paving the way for more sophisticated and reliable constitutive models for other soils and environmental conditions. The ability to directly link microscopic bond degradation to macroscopic mechanical properties represents a substantial advancement in our understanding of soil behavior and opens new avenues for research into the long-term performance of geotechnical structures under environmental degradation. This research refines DEM for more complex, real-world scenarios to enhance predictive reliability and broaden its applicability across a diverse range of materials and loading conditions.

## 2. Methodology

### 2.1. Discrete Element Damage Constitutive Model Based on the Bond Rate

In the DEM, a wide range of contact constitutive models are available, with each model designed to target specific macroscopic material characteristics [41]. Establishing the mapping relationship between the contact constitutive parameters and macroscopic mechanical properties of materials is the basis for accurately predicting the microscopic parameters of materials. This study focuses on compacted loess and the linear contact bond model (LCBM) is adopted based on triaxial test data.

LCBM, introduced by Potyondy [44] in 2004, extends the linear contact model by incorporating bond behavior between contacting particles. The linear contact model itself represents the interaction between two particles as a pair of linear springs, one normal and one tangential to the contact plane. These springs exhibit elastic behavior, with the normal force $F_{i}^{n}$ and tangential force increment $△F_{i}^{s}$ related to the respective displacements $U^{n}$ and $△U_{i}^{s}$ through the following relationships:

(1) $$F_{i}^{n}=K_{n}U^{n}n_{i}$$

(2) $$△F_{i}^{s}=-K_{s}△U_{i}^{s}$$

where

$F_{i}^{n}$ is the normal contact force.

$△F_{i}^{s}$ is the tangential force increment.

$K_{n}$ is the normal stiffness.

$K_{s}$ is the tangential stiffness.

$U^{n}$ is the normal displacement.

$△U_{i}^{s}$ is the tangential displacement increment.

$n_{i}$ represents the contact normal vector.

The stiffnesses $K_{n}$ and $K_{s}$ are calculated from the individual particle stiffnesses $k_{n}$ and $k_{s}$ using parallel spring formulations:

(3) $$K_{n}=\frac{k_{n}^{\left[A\right]}k_{n}^{\left[B\right]}}{k_{n}^{\left[A\right]}+k_{n}^{\left[B\right]}}$$

(4) $$K_{s}=\frac{k_{s}^{\left[A\right]}k_{s}^{\left[B\right]}}{k_{s}^{\left[A\right]}+k_{s}^{\left[B\right]}}$$

where the superscripts *A* and *B* refer to two contacting particles. For the linear contact model, the normal stiffness is considered equal to the normal secant stiffness:

(5) $$k^{n}=\frac{dF^{n}}{dU^{n}}=\frac{d(K^{n}F^{n})}{dU^{n}}=K_{n}$$

LCBM builds upon this linear contact model by introducing tensile and shear bond strengths. These bonds are assumed to exist between contacting particles until the forces exceed these specified strengths. Specifically, there are two types of failure:

Tensile failure: The bond breaks if the normal force exceeds a pre-defined tensile strength *cb_tens*.

Shear failure: The bond breaks if the tangential force exceeds a pre-defined shear strength *cb_shears*.

Upon bond failure, the interaction between the particles reverts to the standard linear contact model described above, with the bond forces set to zero.

**Figure 1:**
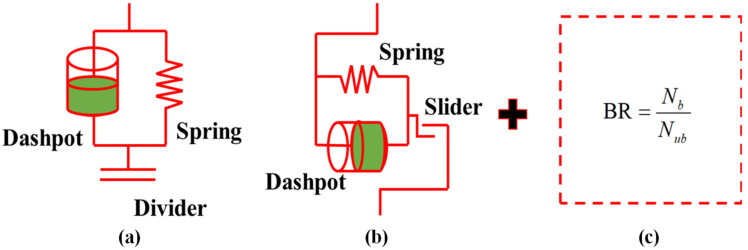
Schematic diagram of a damaged element model considering the bond rate: (**a**) normal component; (**b**) tangential component; (**c**) damage term (explained in Equation (6)).

The bond rate, a key parameter, is introduced to simulate the structural damage of compacted loess under cyclic wet–dry conditions. Figure 1a,b illustrate the conceptual spring models for the normal and tangential components of the LCBM model, respectively, where the dashpot components are optional. Figure 1c presents the newly proposed bond rate parameter component.

The software PFC 6.0 was used in this study. It is important to note that the methodology proposed in this study is general in nature and is not confined to the PFC environment. In the Particle Flow Code (PFC) environment, the macroscopic behavior of materials is directly influenced by the accurate setting of microscopic parameters [45]. However, the influence of these parameters at the macro scale is currently not fully understood. Although numerous contact constitutive parameters are available in PFC, calibrating these parameters without a clear understanding of their physical significance can hinder the achievement of accurate results [26]. Therefore, before the detailed calibration and analysis of the parameters in the wet–dry cycle triaxial test, this study first quantitatively analyzes the potential macroscopic influence of the microscopic parameters of the contact model. The microscopic parameters discussed in this study comprise the following sections.

(1) Stiffness

In practice, *E_mod_*, the equivalent modulus, and *k_ratio_*, the normal and tangential stiffness ratio, are more commonly used because they account for the influence of particle size on stiffness. *K_n_* and *k_s_* are uniquely determined by *E_mod_* and *k_ratio_*.

(2) Strength

The parameters that control the strength between particles include the friction coefficient *fric*, as well as the normal and tangential strength (tensile and shear strength) *cb_tens* and *cb_shears*.

(3) Bond rate

LCBM can establish bonding between adjacent particles, and bound particle clusters can better simulate the particle structure in loess than independent particles can. However, the presence of numerous pores and cracks in soil renders it unrealistic to rigidly bind all particles together.

In the modeling program, particle bonding can only be controlled by specifying the critical gap, while the bond rate cannot be directly controlled. This article introduces the bond rate parameter as one of the microscopic parameters to be calibrated to characterize the actual bonding state inside the soil and to quantitatively describe the wet–dry cycle process. The bond rate is defined as follows (Figure 1c):

(6) $$\mathrm{BR}=\frac{N_{b}}{N_{ub}}$$

In the formula, *N_b_* represents the total number of bonded contacts, and *N_ub_* represents the total number of contacts.

The bond rate not only numerically describes the proportion of particle-to-particle bonding but also serves as a key factor in quantitatively analyzing structural damage under the influence of wet–dry cycles. In addition to the above parameters, the mechanical properties of materials at the macro scale are closely related to the loading rate, a relationship that also applies to Particle Flow Code (PFC) analysis. It is essential that the loading rate accurately reflects the physical and mechanical properties of the material to effectively simulate numerical experiments using PFC. The selection of the loading rate must maintain a quasistatic state in the specimen during the loading process, thereby avoiding mechanical property distortion caused by excessively rapid loading. This study follows the recommendations made by Cho [46] and incorporates the physical properties of compacted loess to determine the appropriate loading rate, ensuring that the analysis results reliably represent the true behavior of the material.

### 2.2. Model Building and Solution Process

#### 2.2.1. Specimen Modeling

The numerical specimen is generated based on the particle size distribution of compacted loess. Three particle size ranges are considered—0.066–0.048 mm, 0.048–0.024 mm, and 0.024–0.012 mm—with proportions of 17.9%, 61.4%, and 20.7%, respectively. The number of particles for each dry density is determined using Equation (7), which relates the number of particles (*M*) to the specimen area (*A*), two-dimensional porosity (*n*_2*d*_), and average particle diameter (*D*).

(7) $$M=\frac{4A\left(1-n_{2d}\right)}{\pi D^{2}}$$

Different dry densities are simulated by adjusting the initial void ratio, using the relationships presented in Equations (8)–(10). The three-dimensional void ratio (e_3*d*_) obtained from the experimental data is converted to a two-dimensional void ratio (e_2*d*_) using Equations (11)–(13). Table 1 shows the corresponding parameters for various dry densities.

(8) $$e_{0}=\frac{\left(1+w_{0}\right)G_{s}\rho_{w}}{\rho_{0}}-1$$

(9) $$e_{0}=\frac{G_{s}\rho_{w}}{\rho_{d}}-1$$

(10) $$n=\frac{e}{1+e}$$

(11) $$n_{2d}=1-{\left(\frac{1-n_{3d}}{\varepsilon }\right)}^{\frac{2}{3}}$$

(12) $$\varepsilon =\frac{\sqrt{2}}{\sqrt{\pi \sqrt{3}}}+Dr\left[\frac{2}{\sqrt{\pi \sqrt{3}}}-\frac{\sqrt{2}}{\sqrt{\pi \sqrt{3}}}\right]$$

(13) $$D_{r}=\frac{e_{max}}{{emin}_{max}}$$

#### 2.2.2. Boundary Conditions and Servo Control

**Figure 2:**
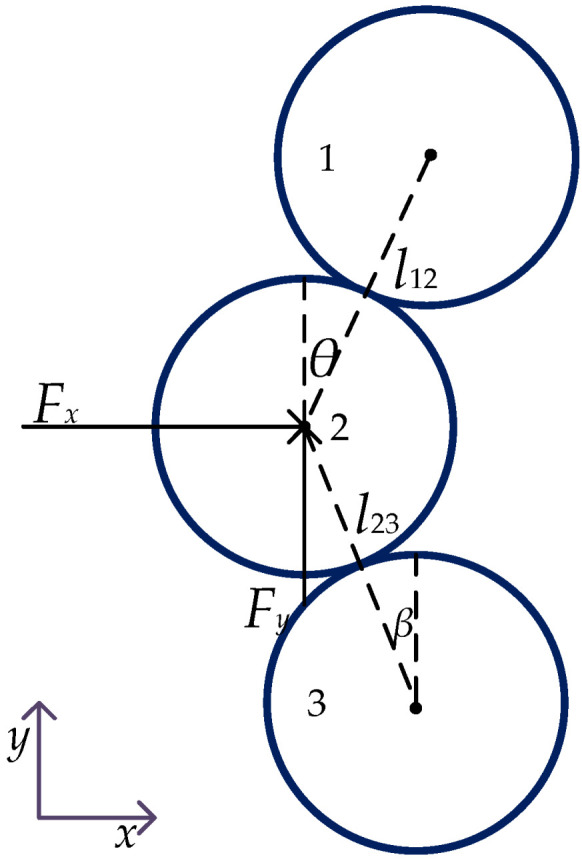
Equivalent force application diagram for flexible particle membrane.

The biaxial test simulation employs a flexible membrane boundary condition. Lateral boundaries consist of chains of bonded particles to mimic a flexible membrane, allowing for lateral deformation under constant confining pressure. The upper and lower boundaries are rigid walls, enabling controlled axial loading through prescribed displacement rates. Confining pressure is maintained through a servo mechanism which adjusts the equivalent concentrated force applied to the membrane particles in each calculation step, as detailed by Equation (8). This differs from a rigid boundary approach where lateral deformation is constrained. Figure 2 shows the definition of the variables in Equation (14).

(14) $$\left\{\begin{array}{c} F_{x}=0.5*(l_{12}\cos\theta +l_{23}\cos\beta )\sigma_{confining}\\ F_{y}=0.5*(l_{12}\sin\theta +l_{23}\sin\beta )\sigma_{confining} \end{array}\right.$$

#### 2.2.3. Loading Process and Solution Procedure

Axial loading is simulated by applying a prescribed velocity to the upper and lower rigid walls. During the simulation, vertical stress and strain are continuously monitored and recorded to construct the stress–strain curve. The dynamic relaxation method, an explicit solver using a central difference scheme, is used to solve the equations of motion This method does not require matrix formation, simplifying calculations and allowing for the consideration of large deformations and nonlinear behavior. The time step is carefully selected to ensure stability and convergence.

## 3. Analysis of Microscopic Parameters

The importance of microscopic parameters in DEM simulation is self-evident, as they serve as a bridge connecting the microscopic behavior between particles and the macroscopic properties of materials. By studying these parameters in detail, the responses of materials under external interference can be captured, and their macroscopic mechanical properties can be predicted. Notably, when the material environment changes, such as during wet–dry cycles, the microscopic parameters directly determine the stability of the material structure and the degree of response during failure.

This section presents an in-depth analysis of the four key microscopic parameters in the damage model: contact stiffness, contact strength, friction coefficient, and bond rate. By changing these parameters and observing their impact on the biaxial test results, the specific effects on the macroscopic mechanical properties can be elucidated. To ensure the stability and reliability of the obtained results, this article focuses on the often-overlooked factor of the loading rate, thereby enhancing the simulation process to more accurately reflect real physical conditions.

### 3.1. Contact Stiffness

To investigate the influence of the contact stiffness parameters on the macroscopic performance of materials in the contact model of PFC, the equivalent modulus *E_mod_* and the stiffness parameters of the modulus ratio *k_ratio_* were selected for analysis based on the LCBM. Biaxial tests were conducted in an unconfined state under different dry density and bond rate conditions, with different values of *E_mod_* and *k_ratio_*.

#### 3.1.1. Equivalent Modulus

This study employs a parametric analysis to investigate the influence of micro-parameters on the macroscopic mechanical behavior of loess. To systematically explore this relationship, several model parameters were assigned values within typical ranges found in the literature, rather than being calibrated to specific experimental data. This approach is justified as the primary objective is to understand the qualitative relationships between micro-parameters and macroscopic responses, not to precisely replicate a specific loess sample’s behavior. The dry density of the soil sample was fixed at 1.7 g/cm^3^. The tensile and shear strengths were both set to 500 kPa, and the friction coefficient was fixed at 0. The bond rate was 100%. The confining pressure was set to 0 kPa, indicating an unconfined condition. *k_ratio_* was fixed at 3.0, and *E_mod_* was varied parametrically at values of 200 kPa, 300 kPa, 400 kPa, and 500 kPa.

**Figure 3:**
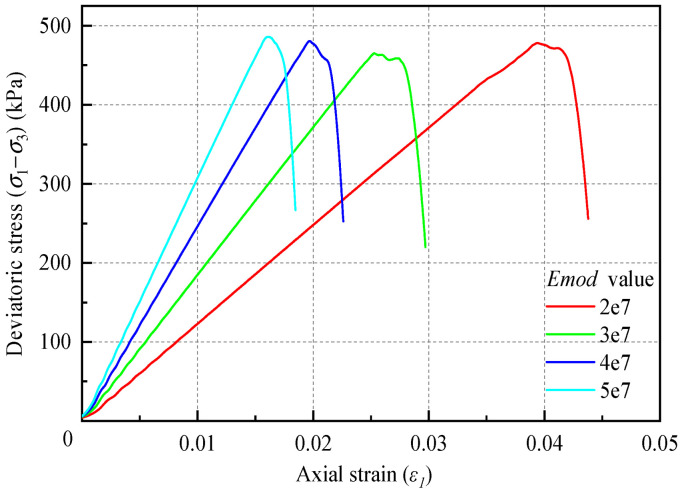
Stress–strain curves of biaxial test simulations with different equivalent modulus values.

**Figure 4:**
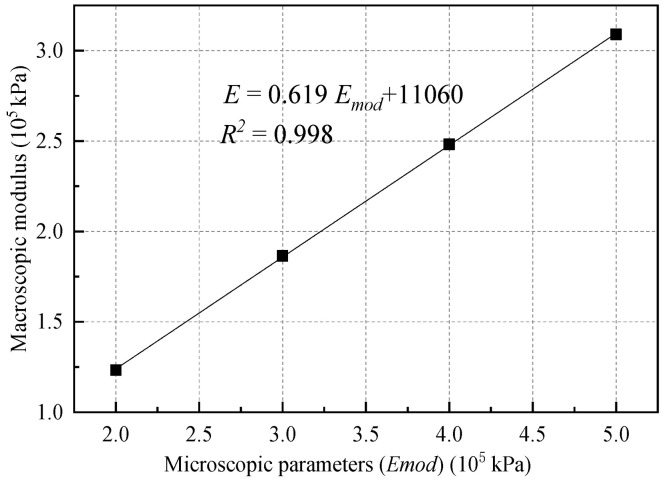
Relationship between equivalent modulus and the macroscopic modulus.

Following the completion of the biaxial test simulations (see Figure 3), the recorded data were fitted to the linear portion of the stress–strain curve. The slope obtained from this fit represents the actual macroscopic modulus of the material. A comparison between the macroscopic modulus (*E*) of the soil and the microscopic equivalent modulus parameter *E_mod_* (see Table 2 and Figure 4) reveals a significant influence of *E_mod_* on the macroscopic modulus of the material, exhibiting a clear linear relationship. However, the value of *E_mod_* does not affect the peak strength of the material. The choice of parameter ranges is based on typical values reported in the relevant literature and aims to provide a comprehensive qualitative analysis of the parameter effects.

When the bond rate is 100%, a clear linear relationship emerges between *E_mod_* and the macroscopic modulus of the soil. This relationship arises from the fact that the numerical specimen is cohesively bonded, resulting in a relatively simple deformation mode prior to the onset of continuous failure. The following sections will discuss the relationship between *E_mod_* and the macroscopic modulus of soil under different bond rates.

**Figure 5:**
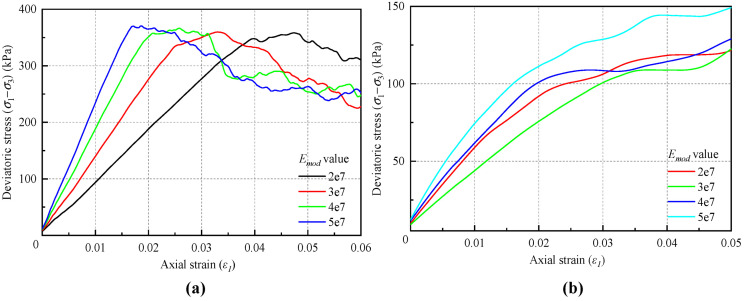
Stress–strain curves for different equivalent modulus values: (**a**) bond rate of 80%; (**b**) bond rate of 40%.

**Figure 6:**
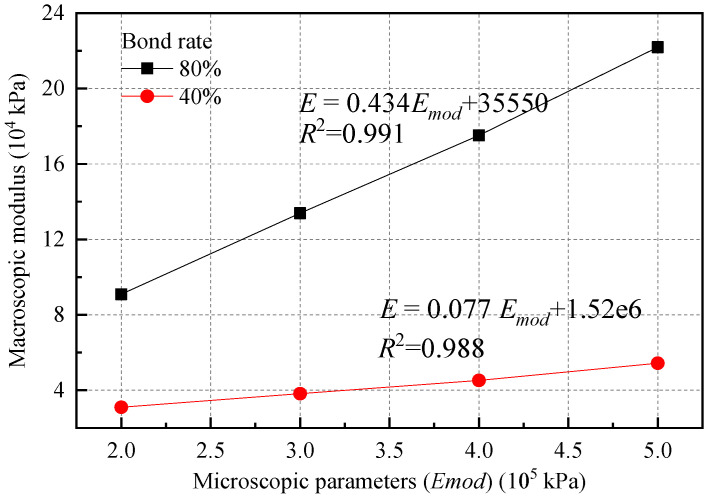
Relationship between equivalent modulus and the macroscopic modulus under different bond rates.

Building on the previous setting of a bond rate of 100%, numerical experiments were conducted to compare and analyze the effects of different *E_mod_* values under two bond rate conditions of 80% and 40%. The stress–strain curves of the simulation results are shown in Figure 5. The results of the macroscopic strength parameters are summarized in Table 3 and illustrated in Figure 6.

A comparison of the simulation results for bond rates of 100%, 80%, and 40% indicates that the macroscopic modulus increases linearly with rising values of the microscopic parameter *E_mod_*. However, as the bond rate decreases, the influence of *E_mod_* on the macroscopic modulus gradually weakens, and the magnitude of the macroscopic modulus also decreases with the bond rate. This phenomenon occurs because a decrease in bond rate leads to a greater proportion of total deformation being attributed to the relative dislocation between soil particles. Furthermore, the variability in bond rates results in the stress–strain curve exhibiting a degree of discreteness. As the bond rate decreases, the stress–strain curve transitions from a strain softening behavior to a strain hardening behavior, a pattern that parallels the changes observed in the stress–strain curve when the dry density of the soil is reduced. When the bond rate is high, the peak strength tends to overshadow the effects of *E_mod_* on soil strength. However, as the soil transitions into strain hardening mode, *E_mod_* significantly influences the material’s strength.

#### 3.1.2. Normal and Tangential Stiffness Ratio

The normal and tangential stiffness ratio *K_ratio_* is defined as the ratio between *Kn* and *K_s_*, that is, *K_ratio_ = K_n_/K_s_*.

**Figure 7:**
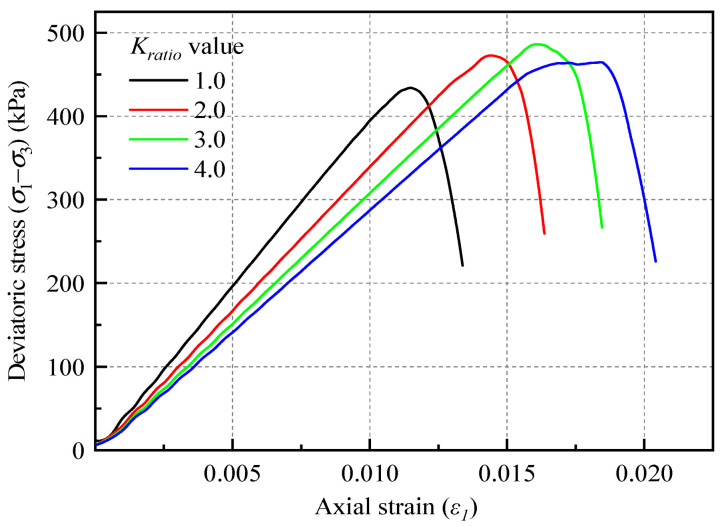
Stress–strain curves for different normal and tangential stiffness ratio values.

According to Figure 7 and Table 4, the *K_ratio_* value exerts a relatively minor influence on the macroscopic modulus of the soil when compared to the *E_mod_* value. The value of *K_ratio_* primarily affects Poisson’s ratio of the soil, which is not pertinent to the focus of this study. Therefore, for subsequent investigations, the value of *K_ratio_* will be fixed at 3.0.

### 3.2. Contact Strength

The contact strength parameters include the normal tensile strength parameter *cb_tens* and the tangential shear strength parameter *cb_shears*. Biaxial tests were conducted to analyze the results of the biaxial tests by proportionally changing *cb_tens* and *cb_shears* by changing the value of *cb_shears* and keeping *cb_ tens* and other conditions constant (i.e., changing the ratio of tensile and shear strength) to explore the influence of the bond strength parameters on the macroscopic properties of the soil.

#### 3.2.1. Proportional Change

**Figure 8:**
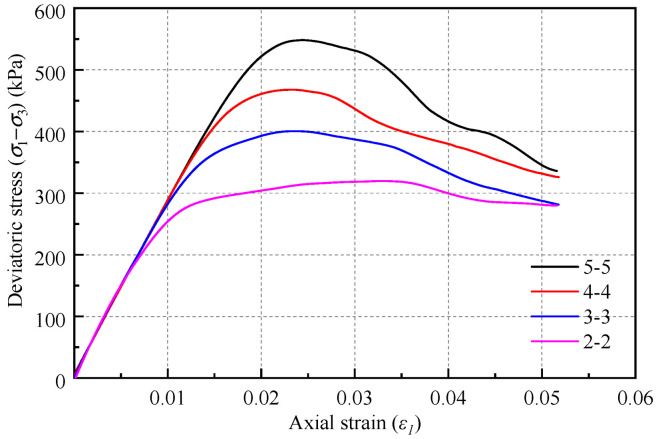
Stress–strain curves under different microscopic strength parameters.

**Figure 9:**
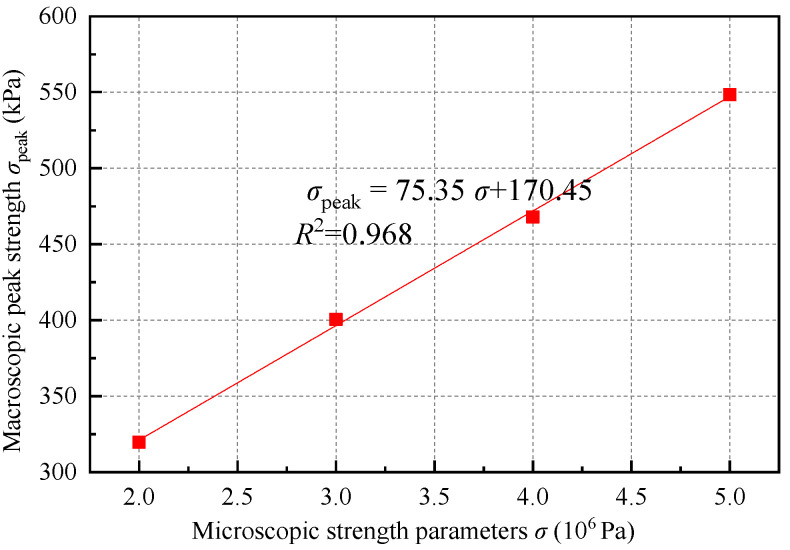
Relationship between microscopic strength parameters and macroscopic peak strength.

Set *cb_tens* and *cb_shears* to 500 kPa, 400 kPa, 300 kPa, and 200 kPa, with the four cases denoted as 5-5, 4-4, 3-3, and 2-2, respectively. The corresponding stress–strain curves of the biaxial test results are illustrated in Figure 8, while the corresponding peak strength results and fitting curves are presented in Table 5 and Figure 9. A clear linear relationship exists between the microscopic strength parameters (*σ*) and the macroscopic peak strength (*σ*_peak_). Furthermore, the microscopic strength parameters do not influence the tangent modulus of the material. Therefore, the impact of these strength parameters can be disregarded during the fitting process of the stiffness parameters.

It is important to note that the linear relationship between the microscopic strength parameters and the macroscopic peak strength, as shown in Figure 9, was derived under the condition that all other parameters were held constant. Consequently, the fitting results should be regarded as a reference for the calibration process and cannot be used to directly infer the microscopic strength parameters from the macroscopic peak strength.

#### 3.2.2. Relative Values

**Figure 10:**
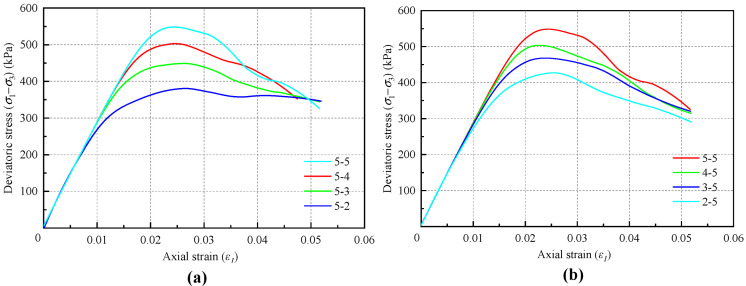
Stress–strain curves: (**a**) under different bond shear strength; (**b**) under different tensile bond strength.

Two situations are considered: (1) The tensile bond strength parameter *cb_tens* is fixed at 500 kPa, while *cb_shears* is set to 500, 400, 300, and 200 kPa. (2) The tensile bond strength parameter *cb_shears* is fixed at 500 kPa, with *cb_tens* varying at 500, 400, 300, and 200 kPa. The numerical biaxial test results are presented in Figure 10.

The influence of the microscopic shear bond strength *cb_shears* on the macroscopic peak strength is significantly greater than that of the microscopic tensile bond strength *cb_tens*. This discrepancy arises because the majority of bond failures during the failure process of the numerical samples occur due to shear failure. When the shear bond strength is varied while keeping the tensile bond strength constant, the macroscopic residual strength shows minimal change. In contrast, alterations in the tensile bond strength do not exhibit this same behavior.

The proportional and relative changes in the bond strength parameters have minimal impact on the macroscopic tangent modulus of the material. In the combination of *cb_shears* and *cb_tens*, *cb_shears* plays a predominant role. To simplify the analysis, the subsequent calibration process will assume that *cb_shears* and *cb_tens* are equal.

### 3.3. Friction Coefficient

The microscopic friction coefficient is not fully equivalent to the macroscopic friction coefficient of the soil, as a bond model was utilized in the numerical modeling process described in this article. Consequently, the mechanical bite effect in the numerical model is lower than in real-world conditions. As a result, the subsequent calibration of the friction coefficient will account for both the material friction strength and the mechanical bite effect, potentially resulting in a friction coefficient that is greater than or equal to 1. Additionally, during the implementation of the bond rate, the bond distance (gap) parameter was set to be too large, leading to increased spacing between the spheres in the model and subsequently raising the friction coefficient.

**Figure 11:**
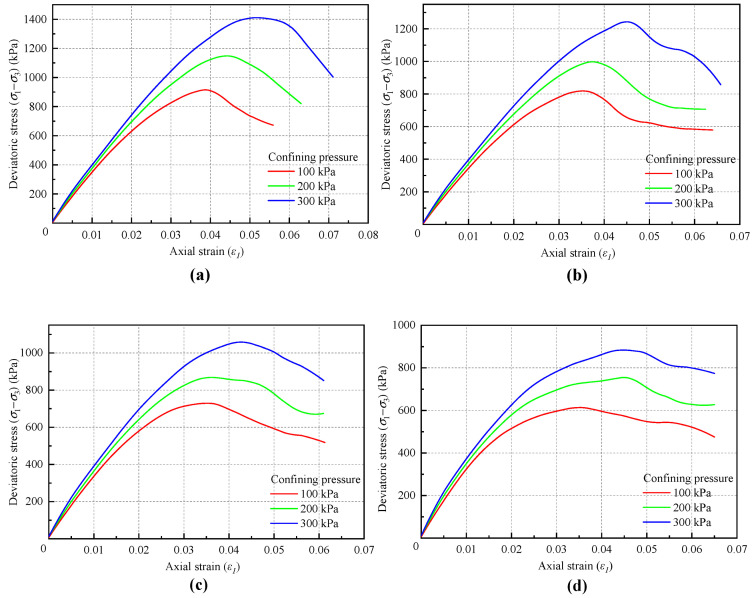
Stress–strain curves under different confining pressure: (**a**) friction coefficient of 0.8; (**b**) friction coefficient of 0.6; (**c**) friction coefficient of 0.4; (**d**) friction coefficient of 0.2.

Figure 11 illustrates the stress–strain curves under different friction coefficients, *fric*. The value of *fric* has minimal impact on the initial portion of the stress–strain curve but has a certain influence on the morphology of the subsequent parts. This occurs because, in the contact bond model, the friction coefficient becomes effective only when the bonding between particles is broken, resulting in little change in the initial part of the curve. However, the effect of *fric* gradually becomes apparent after the number of broken contacts increases.

**Figure 12:**
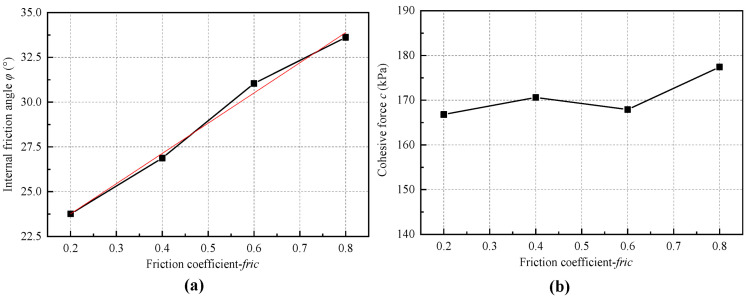
Relationship between the microscopic friction coefficient and the macroscopic (**a**) internal friction angle and (**b**) cohesion force.

Table 6 and Figure 12 show the influence of *fric* on the macroscopic cohesion and internal friction angle. The *fric* value directly determines the internal friction angle, while its impact on cohesion can be ignored.

### 3.4. Bond Rate

This article introduces the concept of the bond rate to describe the weakening of bond strength resulting from the development of pore structures and the dilution effect of water on the bound material during wet–dry cycling. Prior to calibration, the influence of the bond rate on the macroscopic mechanical behavior of the materials was investigated.

The bond rate (BR) parameter, introduced to represent the progressive degradation of inter-particle bonds due to wet–dry cycles, was not directly calibrated using a formal optimization algorithm like nonlinear least squares or a simplex method. Instead, a simple trial-and-error calibration approach was employed. This was due to the study’s focus on the qualitative impact of BR variation on the macroscopic stress–strain response, rather than on precise quantitative fitting to specific experimental data. The objective was to investigate the trends of the stress–strain curves with variations in bond rate, to reflect the deterioration process.

**Figure 13:**
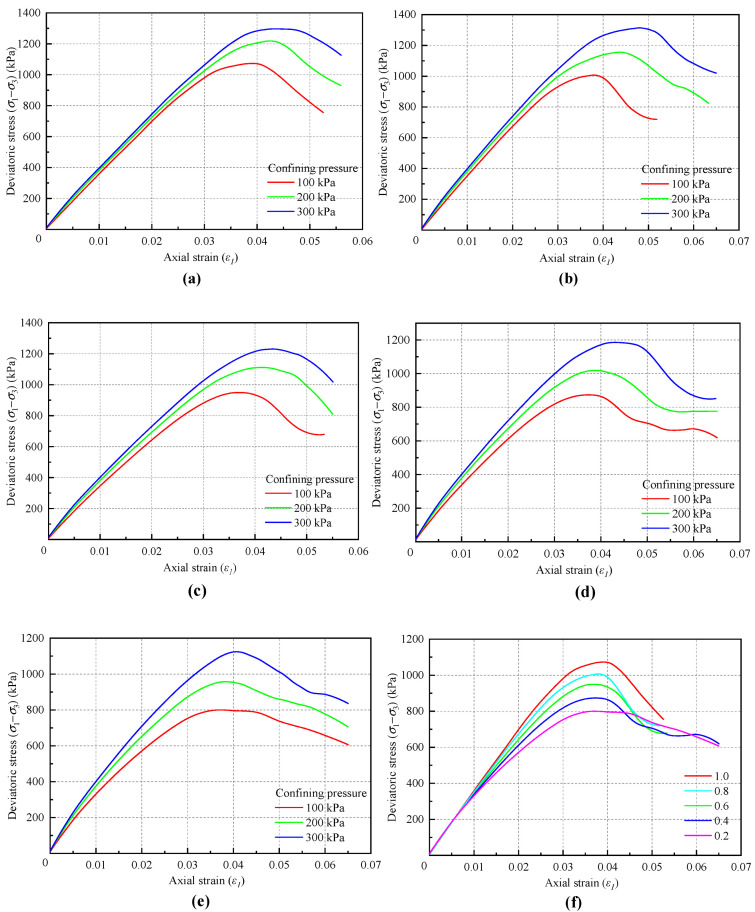
Stress–strain curves under different confining pressure: (**a**) Bond rate = 1.0. (**b**) Bond rate = 0.8. (**c**) Bond rate = 0.6. (**d**) Bond rate = 0.4. (**e**) Bond rate = 0.2. (**f**) Comparison of curves under different bond rates.

**Figure 14:**
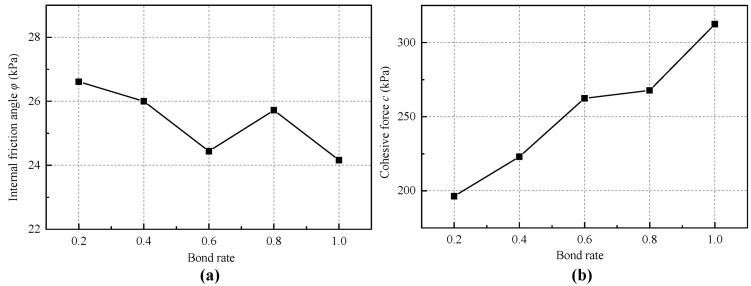
Relationship of bond rate and (**a**) internal friction angle; (**b**) cohesion force.

In this section, numerical biaxial tests were conducted with five bond rates of 1.0, 0.8, 0.6, 0.4, and 0.2. The corresponding stress–strain curves and their comparison are shown in Figure 13. Additionally, Table 7 and Figure 14 show the macroscopic strength parameter results obtained with different bond rates.

Figure 13 illustrates that the influence of the bond rate on the stress–strain curve is relatively minor under high confining pressure, while it has a significant effect under low confining pressure. As the bond rate decreases, the stress–strain curve at low confining pressure exhibits a clear trend toward strain hardening. This occurs because, with constant bond strength, the bond rate dictates the peak strength of the material. A decrease in the bond rate leads to a reduction in peak strength and an increase in the proportion of friction strength, resulting in a change in the shape of the stress–strain curve. Conversely, as the confining pressure increases, the proportion of friction strength relative to total strength also rises, which diminishes the influence of the bond rate. Table 7 and Figure 14 demonstrate that while the bond rate significantly affects the macroscopic cohesion of the material, its impact on the internal friction angle can be considered negligible.

### 3.5. Loading Rate

The analysis process involved five different loading rates: 0.1 m/s, 0.05 m/s, 0.02 m/s, 0.01 m/s, and 0.005 m/s. The material contact parameter settings are shown in Table 8.

**Figure 15:**
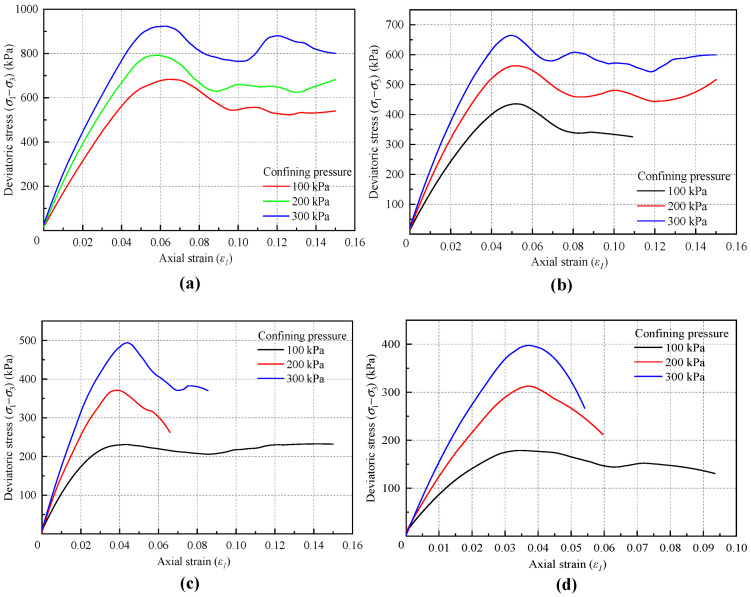

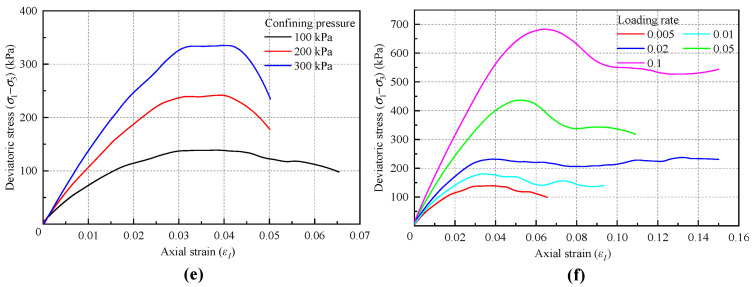
Stress–strain curves under different confining pressure: (**a**) Loading rate = 0.1 m/s. (**b**) Loading rate = 0.05 m/s. (**c**) Loading rate = 0.02 m/s. (**d**) Loading rate = 0.01 m/s. (**e**) Loading rate = 0.005 m/s. (**f**) Comparison of curves under different loading rate.

Figure 15 presents the stress–strain curve results from the biaxial tests conducted at different loading rates, along with a comparison of these results. The comparison reveals that as the loading rate gradually decreases, the shapes of the stress–strain curves under various confining pressures become increasingly similar to the actual test results.

**Figure 16:**
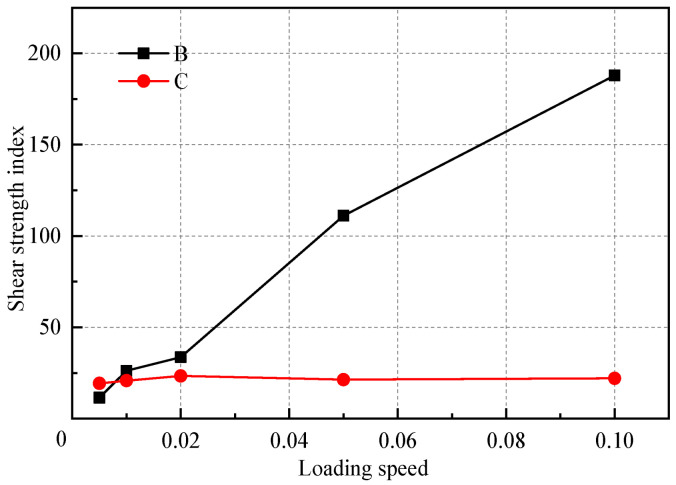
Effect of loading rate on the shear strength index.

Table 9 and Figure 16 provide a comparison of the shear strength indicators obtained at different loading rates and a comparison of computational costs. The results indicate that the loading rate has a significant impact on the cohesive force component of the macroscopic strength index results of the biaxial test, while its impact on the internal friction angle component is relatively small. When the loading rate is below 0.02 m/s, further reductions in the loading rate have a diminishing effect on the results. Considering the computational costs in terms of time, this study establishes the loading rate at 0.02 m/s. This choice balances the need for calculation accuracy with the goal of minimizing computational costs and enhancing calculation efficiency.

### 3.6. Macro Significance of Microscopic Parameters

Based on the analysis of the aforementioned microscopic parameters, the relationships between the microscopic contact parameters and the macroscopic strength parameters can be summarized as follows:

(1) The selection of the loading rate affects both the accuracy of the simulation results and the efficiency of calculations. An excessively high loading rate can lead to an incorrect shape of the stress–strain curve shape and can overestimate the peak strength, whereas a loading rate that is too low can result in unacceptably lengthy computation times. After comprehensive consideration, this study has opted for a loading rate of 0.02 m/s.

(2) The ratio of normal stiffness to tangential stiffness affects the Poisson’s ratio of the material; however, this effect is minimal for the study presented in this paper. Therefore, this ratio was fixed at 3.0 during the analysis.

(3) The proportional and relative changes in bond strength parameters have a negligible impact on the macroscopic tangent modulus of the material. Among the parameters, *cb_shears* and *cb_tens*, *cb_shears* play a dominant role. Therefore, to simplify the analysis process, the calibration process assumed that *cb_shears* and *cb_tens* are equal.

(4) For the strain softening stress–strain curve (reaching a peak strength before 15% strain), the bond strength and bond rate jointly determine the strength. For the strain hardening type (there is no peak before 15% strain, and the strength is taken as the stress at 15% strain), the bond strength, bond rate, friction coefficient, and contact stiffness modulus jointly determine the form of the stress–strain curve, thereby determining the strength of the material.

(5) The majority of the stress–strain curves obtained in the triaxial tests of compacted loess after undergoing wet–dry cycles completed in this article are strain hardening types, so the bond strength, bond rate, friction coefficient, and contact stiffness modulus are all included as parameters to be calibrated in the analysis process.

## 4. Model Applications

In Section 3, we provided a detailed analysis of how each microscopic parameter influences the macroscopic behavior of the material. Subsequently, the theoretical understanding was then applied to the PFC model. Through the empirical analysis in Section 4, with calibration of the bond rate, further understanding of the interaction between the parameters and the impact of wet–dry cycles on the material properties was achieved.

**Figure 17:**
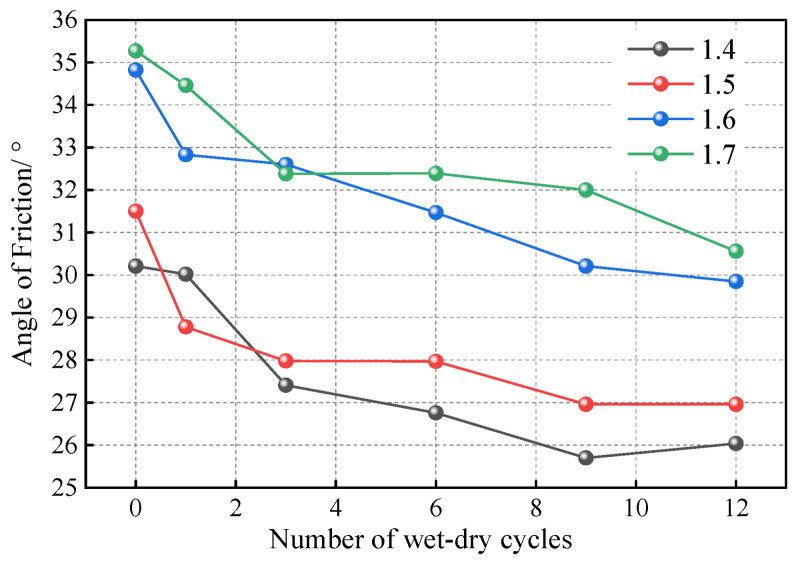
Resulting angle of friction of the test.

**Figure 18:**
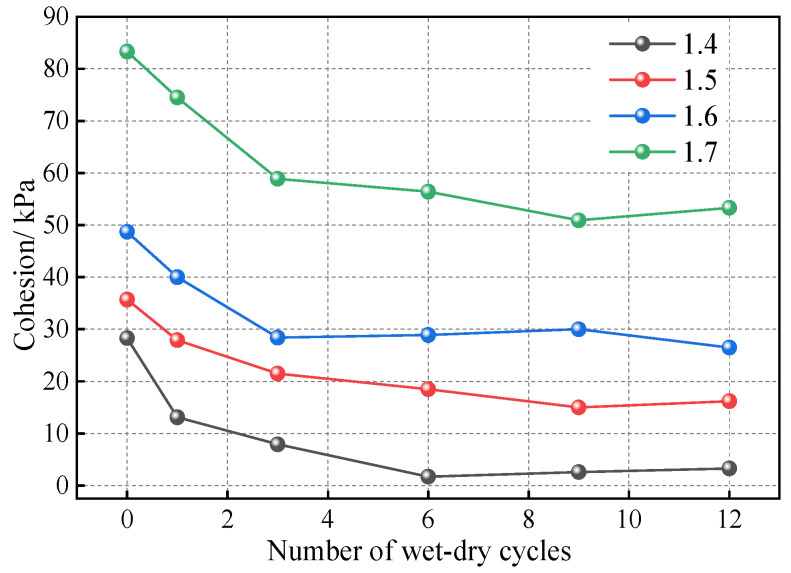
Resulting cohesion of the test.

**Figure 19:**
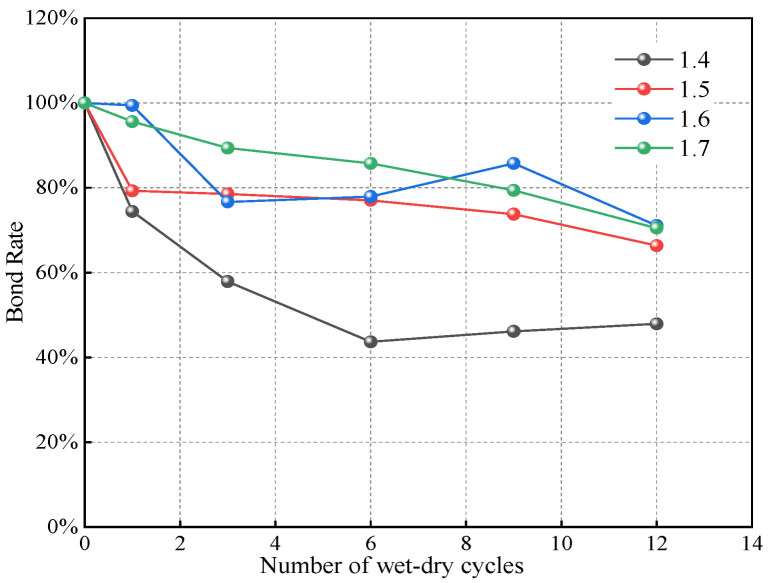
Results of bond rate calibration.

**Figure 20:**
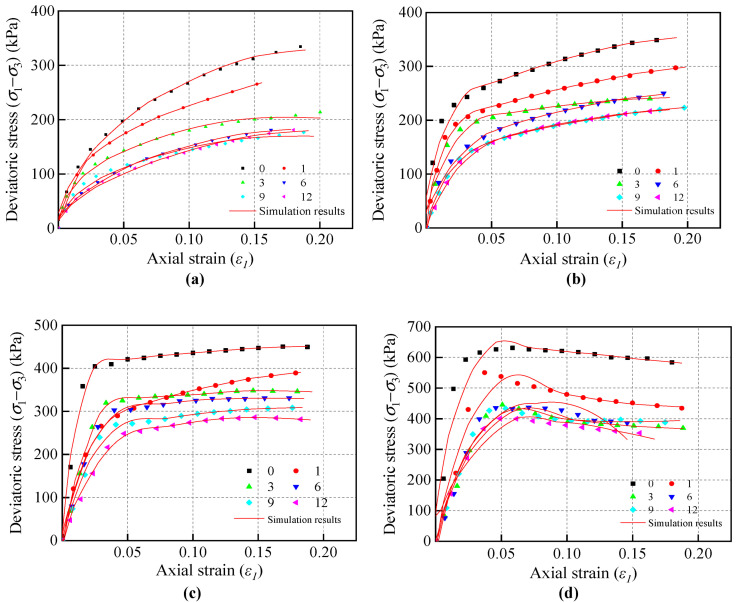
Stress–strain curves of soil samples with various dry densities under different numbers of wet–dry cycles: (**a**) *ρ* = 1.4 g/cm^3^; (**b**) *ρ* = 1.5 g/cm^3^; (**c**) *ρ* = 1.6 g/cm^3^; (**d**) *ρ* = 1.7 g/cm^3^.

To test the proposed method, we conducted a series of wet–dry cycle experiments on compacted loess with various dry densities and calibrated the results using a trial-and-error approach. The test results are shown in Figure 17, Figure 18 and Figure 19. Table 10 and Figure 20 present the resulting of bond rates after the calibration process. According to the definition, the bond rate of the soil sample with 0 wet–dry cycles (i.e., without undergoing wet–dry cycles) is 100%, and after experiencing wet–dry cycles, the bond rate is reduced to varying degrees. As the number of wet–dry cycles increase, the bond rate continuously decreases and gradually stabilizes. Notably, lower dry densities exhibit a more pronounced reduction in bond rate. Specifically, the soil sample with a dry density of 1.4 g/cm^3^ experiences the greatest decrease in bond rate, while the other three soil samples with different dry densities show similar results. This suggests that higher dry densities mitigate the deterioration of the bonding characteristics due to wet–dry cycles. There appears to be a critical dry density threshold: when the dry density exceeds this critical value, further increases do not significantly enhance resistance to bonding deterioration. The simulation results after the calibration of the bond rate are shown in Figure 20, and satisfactory results can be observed.

## 5. Discussion

This study presents a novel DEM model for compacted loess, incorporating a bond rate parameter to simulate constitutive damage behavior under cyclic wet–dry conditions. The introduction of this parameter allows for a more realistic representation of the degradation process than previous models, which often lack a direct quantitative measure of bond deterioration. The model’s ability to link microscopic bond degradation to macroscopic mechanical properties is a significant advancement, providing valuable insights into the micro–macro relationship in loess. The results demonstrate a clear relationship between the bond rate and the macroscopic mechanical properties of the loess, specifically its cohesion, under different confining pressures. A decrease in bond rate leads to a reduction in macroscopic strength, particularly under low confining pressures, where the influence of the bond rate is more pronounced.

The systematic parameter sensitivity analysis clarifies the influence of various microscopic parameters (contact stiffness, contact strength, friction coefficient, and bond rate) on the macroscopic mechanical behavior. This analysis highlights the importance of considering all these parameters when modeling loess behavior, and especially under environmental degradation. For instance, the analysis shows that while the equivalent modulus significantly influences the macroscopic modulus, the effect is lessened at lower bond rates. Similarly, shear strength plays a more dominant role than tensile strength in determining macroscopic peak strength. The optimal loading rate was also determined by analyzing its influence on stress–strain curves and computational efficiency.

The calibrated bond rate values, demonstrating a decrease with increasing wet–dry cycles and a stronger effect at lower dry densities, support the model’s ability to realistically capture the effects of environmental degradation. The existence of a critical dry density, above which further increases in dry density offer minimal additional resistance to bond deterioration due to wet–dry cycles, is also a valuable finding. This suggests potential strategies for improving the long-term performance of compacted loess structures.

The limitations of the current study should also be acknowledged. While the model effectively captures the effects of wet–dry cycles on loess strength, further research is needed to validate its applicability to other soils and loading conditions. The qualitative calibration approach for the bond rate parameter could be improved with the implementation of a formal optimization technique in future work. The two-dimensional nature of the model also presents a limitation for fully capturing three-dimensional soil behavior.

Despite these limitations, the model presents a significant advancement in DEM modeling of loess behavior under environmental degradation. The introduction of the bond rate parameter provides a more accurate and physically meaningful representation of damage progression, improving the prediction of material properties and aiding the design of safer and more durable earth structures. Future work could explore incorporating more complex failure criteria and coupling the model with other numerical methods to further refine its predictive capability and expand its applicability.

## 6. Conclusions

This study presents a novel DEM model for compacted loess, incorporating a “bond rate” parameter to quantify the impact of wet–dry cycles on its mechanical properties. This represents a significant advancement in our understanding of the micro–macro relationship in loess subjected to environmental degradation. Our findings demonstrate a clear correlation between the bond rate and macroscopic strength, particularly under lower confining pressures. The systematic parameter sensitivity analysis highlights the critical roles of the contact stiffness, strength, friction coefficient, and bond rate in determining macroscopic behavior. The calibrated bond rate values reveal a significant decrease with increasing wet–dry cycles, more pronounced at lower dry densities, and the existence of a critical dry density threshold beyond which further increases offer minimal additional resistance to degradation. This improved understanding of loess behavior under environmental stress has significant implications for the design and safety of earth structures.

Future research will focus on several key areas. First, we will validate the model’s applicability to a wider range of soils and loading conditions, enhancing its predictive capabilities beyond compacted loess. Second, we will refine the model by incorporating more sophisticated failure criteria and potentially coupling it with other numerical methods for a more comprehensive representation of soil behavior. Third, we plan to investigate the potential for optimizing loess microstructure to enhance its resistance to degradation under cyclic wet–dry conditions. These future studies will further advance our understanding of soil mechanics and contribute to improved geotechnical engineering design practices.

## Figures and Tables

**Table 1:** Soil sample dry density and porosity parameter comparison table.

Dry Density (g/cm^3^)	3D Void Ratio *e*_3*d*_	3D Porosity *n*_3*d*_	2D Porosity *n*_2*d*_	Particle Number
1.4	0.94	0.49	0.23	6397
1.5	0.81	0.45	0.22	6547
1.6	0.70	0.41	0.20	6710
1.7	0.60	0.38	0.19	6880

**Table 2:** Microscopic equivalent modulus and corresponding macroscopic modulus.

Microscopic *E_mod_* (10^5^ kPa)	2	3	4	5
Macroscopic modulus (10^5^ kPa)	1.23	1.86	2.48	3.09

**Table 3:** Comparison of macroscopic strength parameters under two bond rate conditions.

Microscopic *E_mod_* (10^5^ kPa)	2	3	4	5
Macroscopic modulus (80%) (10^5^ kPa)	0.9096	1.339	1.751	2.219
Macroscopic modulus (40%) (10^5^ kPa)	0.310	0.383	0.453	0.543

**Table 4:** Equivalent modulus and corresponding macroscopic modulus.

Microscopic *E_mod_* (10^5^ kPa)	1.0	2.0	3.0	4.0
Macroscopic modulus (80%) (10^5^ kPa)	3.982	3.419	3.107	2.903

**Table 5:** Strength combination and corresponding macroscopic modulus.

Strength combination	5-5	4-4	3-3	2-2
Macroscopic peak strength (kPa)	548.4	468.0	400.6	319.7

**Table 6:** Comparison of macroscopic strength parameters under different friction coefficients.

Bond rate	0.2	0.4	0.6	0.8
Cohesive force (kPa)	166.8	170.6	167.9	177.4
Internal friction angle (°)	23.76	26.88	31.04	33.62

**Table 7:** Comparison of macroscopic strength parameters under different bond rates.

Bond rate	0.2	0.4	0.6	0.8	1.0
Cohesive force (kPa)	196.4	223.0	262.4	267.7	312.4
Internal friction angle (°)	26.61	26	24.44	25.72	24.16

**Table 8:** Detailed parameter settings.

Microscopic Parameters	*E_mod_* (10^5^ kPa)	*K_ratio_*	*cb_tens* (10^3^ kPa)	*cb_shears* (10^3^ kPa)	*fric*
Value	5	3.0	2	2	0.6

**Table 9:** Comparison of shear strength indicators and calculation times under different loading rates.

Loading Rate (m/s)	Cohesive Force (kPa)	Internal Friction Angle (°)	The Fit Correlation Coefficient R^2^	PFC Calculation Time (min)
0.1	187.9	22.1	0.9996	11
0.05	111.1	21.35	0.9991	19
0.02	33.7	23.36	0.9997	29
0.01	26.2	20.79	0.9996	59
0.005	11.5	19.29	0.9992	128

**Table 10:** Calibration results of the bond rate.

Number of Wet–Dry Cycles	Dry Density (g/cm^3^)			
	1.4	1.5	1.6	1.7
0	100%	100%	100%	100%
1	74.42%	79.29%	99.41%	95.57%
3	57.90%	78.54%	76.66%	89.36%
6	43.68%	77.05%	77.93%	85.73%
9	46.16%	73.77%	85.73%	79.38%
12	47.90%	66.36%	71.10%	70.50%

## Data Availability

The original contributions presented in the study are included in the article, further inquiries can be directed to the corresponding author.
